# Adjusting to duty hour reforms: residents’ perception of the safety climate in interdisciplinary night-float rotations

**Published:** 2018-11-12

**Authors:** Alexandre Lafleur, Adrien Harvey, Caroline Simard

**Affiliations:** 1 Département de médecine, Faculté de médecine, Université Laval, Québec, Canada; 2 QMA-CMA-MD Educational Leadership Chair in Health Professions Education, Vice-décanat à la pédagogie et au développement professionnel continu, Faculté de medicine, Université Laval, Québec, Canada

## Abstract

**Background:**

New scheduling models were needed to adjust to residents’ duty hour reforms while maintaining safe patient care. In interdisciplinary night-float rotations, four to six residents from most residency programs collaborated for after-hours cross-coverage of most adult hospitalised patients as part of a Faculty-led rotation. Residents worked sixteen 12-hour night shifts over a month.

**Methods:**

We measured residents’ perception of the patient safety climate during implementation of night-float rotations in five tertiary hospitals. We surveyed 267 residents who had completed the rotation in 2015-2016 with an online version of the Safety Attitudes Questionnaire. First year residents came from most residency programs, second- and third-year residents came from internal medicine.

**Results:**

One-hundred-and-thirty residents completed the questionnaire. Scores did not differ across hospitals and residents’ years of training for all six safety-related climate factors: teamwork climate, job satisfaction, perceptions of management, safety climate, working conditions, and stress recognition.

**Conclusion:**

Simultaneous implementation in five hospitals of a Faculty-led interdisciplinary night-float rotation for most junior residents proved to be logistically feasible and showed similar and reassuring patient safety climate scores.

## Abstract (fr)

**Contexte:**

De nouveaux horaires de garde en établissements hospitaliers étaient nécessaires pour s’adapter aux réformes des heures de travail des résidents tout en maintenant des soins sécuritaires pour les patients. Dans les stages cliniques de nuit interdisciplinaires, quatre à six résidents de la plupart des programmes de résidence ont collaboré pour assurer une couverture croisée, après les heures normales de travail, de la plupart des patients adultes hospitalisés. Les résidents ont travaillé seize nuits de 12 heures durant un mois.

**Méthodes:**

Nous avons mesuré la perception des résidents du climat de travail lié à la sécurité des patients lors de la mise en place de stages de nuit dans cinq hôpitaux universitaires. Nous avons interrogé 267 résidents ayant terminé le stage en 2015-2016 avec une version numérique du *Safety Attitudes Questionnaire*. Les résidents de première année provenaient de la plupart des programmes de résidence, les résidents de deuxième et troisième années provenaient du programme de médecine interne.

**Résultats:**

130 résidents ont complété le questionnaire. Les scores ne différaient pas entre les hôpitaux et les années de formation des résidents pour les six facteurs liés à la sécurité des patients: climat de travail en équipe, satisfaction au travail, perceptions des supérieurs, climat de sécurité, conditions de travail et reconnaissance du stress.

**Conclusions:**

La mise en place simultanée, dans cinq hôpitaux, de stages cliniques de nuit réunissant des résidents juniors de la majorité des programmes de résidence fut logistiquement possible et a montré des résultats similaires et rassurants sur le climat de travail lié à la sécurité des patients.

## Introduction

Duty hour reforms for medical residents were driven by the expectation that new on-call schedules would provide better conditions for learning and safer patient care.^1^ However, systematic reviews of the last decade of duty hour reforms did not find convincing evidence of improved patient safety.^1-3^ Some studies in acute care and surgery settings even raised concerns regarding an increased morbidity and mortality after the implementation of new scheduling systems.^4-7^

A 16-hour workday restriction has been in effect in the Province of Québec (Canada) since 2012 and night calls are limited to twelve hours.^8-10^ Replacing 24-hour on-call periods, new scheduling models were needed to address both patient safety and educational standards.^10-12^ At our institution, due to mandatory pre- and post-call absence, most of these scheduling models led to fifty percent workday attendance at clinical duties and a catastrophic discontinuity in clinical and educational activities.

To overcome this challenge, we implemented an innovative model of interdisciplinary night-float rotation.^13^ For four consecutive weeks, we grouped three to six residents from most residency programs to work as teams for after-hours consultations and cross-coverage of hospitalised patients.^13^ We used the term “float” because residents shared the workload of multiple services and “rotation” because it was scheduled in the residency curriculum and assessed like other mandatory clinical rotation.

Some raised legitimate concerns regarding the working climate resulting from this unusual combination of residents from different backgrounds working in a new environment with supervisors from all disciplines and providing coverage for almost all hospitalised patients. A positive working climate ensuring residents’ wellbeing and patient safety being at the origin of duty hour reforms, residents’ perception in that regard was a priority of the quality assurance process.^10^

The Safety Attitudes Questionnaire (SAQ) was benchmarked in multiple care units, including general inpatient and acute care settings, and studied in association with patient outcomes.^14-18^ Its goal is to elicit a snapshot of the safety culture through surveys of frontline worker perceptions.^15^ Within the first fourteen months of implementation of our night-float rotations and using the SAQ we measured residents’ attitude regarding patient safety in five hospitals.

## Methods

### Setting

We implemented night-float rotations simultaneously in five tertiary hospitals of Université Laval in Québec City, Canada (1500 hospitalised patients). In each site, night-float teams gathered two to four first-year residents and one or two second- or third-year internal medicine residents. Residents worked together to ensure cross-coverage of adult patient in-house calls, including new consultations at the emergency room (all sites), on most medical and surgical hospitalisation units (all sites) and in some intensive care units (sites 2 and 4). Residents worked under the immediate supervision of senior residents and on-call physicians depending on the speciality involved in each case. Residents were assigned to 16 shifts over four consecutive weeks. They worked Monday to Thursday from 8 p.m. to 8 a.m. and were exempted from daytime activities. University personnel managed residents’ distribution in collaboration with residency programs.

Night-float rotations were mandatory for first-year residents from most residency programs. Instructional methods comprised video recordings, online resources, reflexive activities and logbooks. For each period, in each site, a clinical supervisor recruited among all participating specialities was responsible for residents’ follow-up and assessment. Group meetings with supervisors took place weekly. Assessment criteria focused on collaboration, professionalism and recognition of medical and surgical emergencies.

### Population

We surveyed by email, with two reminders, all 267 residents who had completed the night-float rotation from August 2015 to November 2016. First year residents came from most residency programs, second- and third-year residents from internal medicine.

### Measurement of patient safety climate

With permission from the authors, we translated the short form of the SAQ in Canadian-French (SAQ-FR), confirmed by four bilingual educators.^19^ The questionnaire was translated back to English electronically and all sentences maintained their semantic properties. Completed online, all questions began with “In your last night-float rotation […].” Thirty-one items of the 36-item SAQ elicit residents’ attitude toward six safety-related climate factors (five filler items).^15^ Answers were given on a Likert scale of 1 to 5: 1=disagree strongly, 2=disagree slightly, 3=neutral, 4=agree slightly, 5=agree strongly. In accordance with Sexton *et al*.^15^ mean scores for each of the six factors were reported on a 100-point scale using this formula: (mean score of the items of a factor – 1)*25.

### Patient safety climate analysis

We calculated differences between residents’ years of training and between sites with multivariate analysis of variance (MANOVA) to consider simultaneously the six dimensions of the SAQ.^15,18^ As the sample is fairly small and groups unequal, Pillai’s trace was preferred for its robustness.^20^ We used *Post-hoc* Bonferroni tests to investigate significant differences among groups. We performed reliability analyses accordingly to classical test theory, hence we conducted item analysis to obtain Cronbach’s *alpha* as internal consistency coefficients and corrected item-total correlations as discrimination indices. We used IBM SPSS statistics for Windows Version 20 (IBM Corp., Armonk, NY, USA).

The Research Ethics Committee of Laval University, applying rule 2.5 for quality improvement of educational projects, waived ethical approval. All participants were provided a consent form before completing the questionnaires, which remained anonymous.

## Results

As presented in Table 1, 130 residents voluntarily participated in the study (48% response rate) and none were excluded. All surveys were completed without missing data and hence included for analysis. Descriptive statistics for the whole sample, across hospitals, and across residents’ years of training are summarized in Table 2.

**Table 1:** Baseline characteristics of respondents

Characteristics	Total, no. (%) n = 130
**Gender**	
Female	72 (55.4 %)
Male	58 (44.6 %)
**Age Group (years)**	
20-24	44 (33.8 %)
25-29	68 (52.3 %)
30-34	9 (6.9 %)
35 or more	9 (6.9 %)
**Speciality^a^**	
Internal Medicine	58 (44.6 %)
Family Medicine	28 (21.5 %)
Psychiatry	13 (10.0 %)
Radiology	8 (6.2 %)
Surgical subspecialties	9 (6.9 %)
Other specialities	17 (13.1 %)

*Note. a = The distribution in the population (n=267) was: internal medicine (38.2 %), family medicine (20.2 %), psychiatry (7.9 %), radiology (4.9 %), surgical subspecialties (9.4 %) and other specialities (19.5 %)*.

**Table 2:** Safety attitude questionnaire scores during implementation of night-float rotations in five tertiary hospitals

	Residents’ mean Safety Attitude Questionnaire scores for six safety-related climate factors *Scores on 100-point scales (standard deviations)*					
**Respondents**	**Teamwork climate**	**Safety climate**	**Job satisfaction**	**Stress recognition**	**Perception of management**	**Working conditions**
Perceived quality of collaboration between personnel	Perceptions of a strong and proactive organizational commitment to safety	Positivity about the work experience	How performance is influenced by stressors	Approval of managerial action	Perceived quality of the work environment and logistical support
**All residents** (n = 130)	73.2 (12.2)	62.3 (16.5)	70.8 (19.9)	74.7 (15,6)	71.1 (13.2)	79.8 (12.8)
**Site 1**	71.6 (8.8)	60.6 (12)	67.1 (15.7)	73.1 (15)	68.3 (9.6)	76.9 (11.8)
**Site 2**	78.3 (7.9)	68.8 (11.7)	75.0 (15.5)	77.7 (12.8)	75.4 (9.9)	84.2 (10.1)
**Site 3**	70.7 (15.9)	63.4 (20.7)	69.0 (23)	75.3 (17.5)	73.2 (12.2)	76.8 (14.3)
**Site 4**	72.5 (13.9)	63.0 (16.3)	68.2 (20.5)	72.0 (16.2)	67.0 (13.3)	80.5 (9.6)
**Site 5**	73.5 (11.9)	57.5 (18.1)	74.5 (19.9)	75.6 (16.5)	68.1 (14.8)	80.8 (15.7)
**First-year residents** (n = 88)	72.5 (12.5)	62.5 (17.9)	71.9 (19.9)	75.4 (15.6)	71.0 (12.3)	79.7 (14.3)
**Second- and third-year residents** (n = 42)	74.8 (11.4)	61.9 (13.3)	68.7 (19)	73.4 (15,7)	68.5 (12.5)	80.1 (9.3)
***F*(1, 128)**	1.024	0.273	0.877	0.460	1.650	0.024
***p***	0.313	0.602	0.351	0.499	0.201	0.878

Scores on the SAQ-FR do not seem to vary across training levels or sites. Mahalanobis distance revealed 21 cases being multivariate outliers, which were excluded from analysis. MANOVA across residents’ years of training revealed no significant differences between the first-year and the second- and third-year groups (*p* = .112). As for mean differences between different sites, MANOVA revealed no significant differences between the first-year and second- and third-year groups (*p* = .076). When both considered in the same analysis, the effect of training level*site was not statistically significant either (*p* = .105), nor were the effects of training level (*p* = .202) and site (*p* = .069). However, the sample remained small; hence, statistical power was limited to observe significant differences. When performing individual ANOVAs which require smaller samples but are more risky because type I error is multiplied with each combined ANOVA,^20^ differences between groups were significant for Perception of management between sites (*F*[4,129] = 2.446, *p* = .050), but no Bonferroni test was significant. The ANOVAs on training levels were also non-significant (*p* = .201 to .878). SAQ-FR showed variable internal consistency levels as presented in Table 3, with Cronbach’s α from of .54 for working conditions to .85 for job satisfaction, with .86 for the whole scale.

**Table 3:** SAQ-FR dimensions means (standard deviations), correlations (Pearson’s), and Cronbach’s alpha (n=130)

Factors	Means (SD)	1.	2.	3.	4.	5.	6.
**1. Teamwork climate**	3.93 (0.49)	**.64**					
**2. Safety climate**	3.49 (0.62)	.57^a^	**.75**				
**3. Job satisfaction**	3.83 (0.76)	.60^a^	.45^a^	**.85**			
**4. Stress recognition**	3.99 (0.62)	-.11	-.22^b^	-.08	**.72**		
**5. Perception of management**	3.84 (0.53)	.36^a^	.42^a^	.50^a^	.08	**.64**	
**6. Working conditions**	4.19 (0.51)	.48^a^	.54^a^	.57^a^	-.18^b^	.41^a^	**.54**

Note. a = p < 0.01; b = p < 0.05; Cronbach’s alpha appear in bold on the diagonal

## Discussion

**Figure 1:**
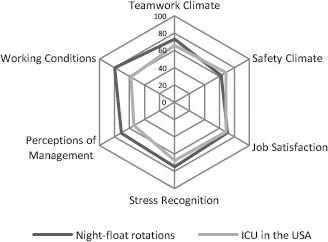
Radar chart of safety attitude questionnaire mean scores from night-float rotations (using SAQ-FR, n=130, 5 sites) and intensive care units in the United States of America (results from Sexton, Helmreich, Neilands *et al*.^15^ using 40-item SAQ, n=3029, 53 sites)

Faculty-led simultaneous implementation in five hospitals of a mandatory interdisciplinary night-float rotation for most junior residents was logistically feasible and showed similar and reassuring patient safety climate scores. Provided for illustrative purposes, Figure 1 contains mean SAQ scores of intensive care units where health professionals are also working at night in stressful environments.^15^ Patient safety climate scores were similar across all five sites for all safety-related climate factors. Those results resonate with survey studies where residents in a night-float system felt an improvement in patient care, reporting that “better care was provided by a rested physician in spite of being less familiar with the patient.”^21,22^

Awaiting confirmation from a qualitative study underway, we believe certain characteristics of our night-float rotation had positive effects on the working climate. For example, the unique interdisciplinary nature of this rotation may be reflected in teamwork climate scores. Also, clear rules were given to residents and hospital personnel regarding call dispatch among the team to regulate the workload. Influencing the perception of management, each team could report to and had scheduled meetings with a supervisor who provided feedback and guidance. Although supervisors were not constantly on-site, all new consultations were discussed immediately with the on-call physician or senior resident. Also, second- and third-year internal medicine residents contributed to role modelling, feedback and bedside teaching, known as key elements for the educational value of night-float assignments by residents and faculty.^23^

### Limitations

The average response rate of SAQ in other studies is 67%.^15^ Subjected to sampling bias, our response rate of 48% should be interpreted as residents’ opinion regarding the safety climate. Response rates over 60% in each night-float teams would be needed for definitive cultural assessment.^14^ Night-float teams were constantly moving from one physical work unit to the other. Therefore, the safety climate was assessed among the night-float teams of residents, who share common management, clinical and operational objectives. We did not include responses from the large number of health professionals in contact with residents during their rotation. Based on previous surveys, residents’ self-assessment may overestimate the quality of care and differ from what would be observed by nurses and supervisors during night floats.^24^ Incompletely tested in the SAQ, night-float rotations, although they offer more stability, can be associated with the sleep and alertness disturbances associated with night work.^25-27^

Night-float rotations provide a strategic setting to study the educational outcomes of the on-call period.^3,^28^,29^ By studying an entire rotation, we gained insight into the hidden curriculum of the on-call period, in particular residents’ positive perception of the learning environment at night.^30^ Data on patients’ outcome and satisfaction with emphasis on continuity of care are needed to triangulate our results.^3,31^ This study does not aim to compare this new scheduling model with previous schedules. However, it provides observational data on a promising model that will draw interest as 12- and 16-hour work limits are applied in other provinces and countries.^10^

## Appendix A Supplementary material

**Short form of the Safety Attitudes Questionnaire translated in Canadian-French and adapted for night-float rotations (SAQ-FR)**

**Table:**

**Teamwork Climate (climat de travail en équipe)**
1. L’avis des résidents est bien reçu par les autres intervenants
2. Il est difficile de dénoncer un problème concernant les soins aux patients que j’aurais pu percevoir
3. Les conflits sont résolus de façon appropriée (par exemple, c’est ce qui est le mieux pour le patient qui prévaut et non pas qui a raison)
4. J’ai le support des autres résidents pour m’occuper des patients
5. Il est facile pour les résidents de poser des questions lorsqu’il y a quelque chose qu’ils ne comprennent pas
6. Les résidents et le personnel infirmier travaillent ensemble comme une équipe bien coordonnée
**Safety Climate (climat de sécurité)**
7. Je me sentirais en sécurité si j’étais traité comme un patient par un résident en stage de nuit
8. Les erreurs médicales sont gérées de façon appropriée
9. Je sais à qui m’adresser directement pour poser mes questions sur la sécurité des patients
10. Je reçois une rétroaction appropriée sur mes performances
11. Il est difficile de discuter des erreurs
12. Je suis encouragé par mes collègues à signaler toute inquiétude sur la sécurité des patients que je pourrais avoir
13. Le climat de travail fait en sorte qu’il est possible d’apprendre des erreurs des autres d’une manière constructive
**Job Satisfaction (satisfaction au travail)**
15. J’apprécie mon travail clinique durant le stage
16. Faire mon stage clinique de nuit dans ce milieu est comme faire partie d’une grande famille
17. C’est un bon milieu pour faire son stage clinique de nuit
18. Je suis fier de travailler dans ce milieu de travail
19. Le moral des résidents durant ce stage est bon
**Stress Recognition (reconnaissance du stress)**
20. Mes performances sont affectées lorsque ma charge de travail devient excessive
21. Je suis moins efficace au travail lorsque je suis fatigué
22. Je suis plus à risque de faire des erreurs dans des situations difficiles ou tendues
23. La fatigue affecte mes performances dans des situations de soins aigus
**Perception of Management (perception des superviseurs)**
24. Mon programme de résidence, le médecin superviseur du stage et les médecins de garde supportent mon travail quotidien
25. Mon programme de résidence, le médecin superviseur du stage et les médecins de garde ne compromettent pas volontairement la sécurité des patients
26. Mon programme de résidence, le médecin superviseur du stage et les médecins de garde font un bon travail
27. Les problèmes interpersonnels sont pris en charge de manière constructive par un superviseur
28. Je reçois au bon moment et de façon adéquate les informations qui pourraient affecter mon travail
**Working Condition (conditions de travail)**
29. Le nombre de résidents durant ce stage était suffisant pour s’occuper de l’ensemble des patients
30. Le milieu hospitalier contribue positivement à la formation des nouveaux résidents en stage de nuit
31. Toute information nécessaire pour les décisions diagnostiques ou thérapeutiques étaient facilement disponibles
32. Les résidents de mon niveau sont adéquatement supervisés
**Others** *(not included in analysis as stated by the scoring key of SAQ)*
14. Mes suggestions sur la sécurité des patients seraient considérées si je les exprimais à un superviseur
33. J’ai eu une bonne collaboration avec le personnel infirmier
34. J’ai eu une bonne collaboration avec les médecins superviseurs
35. J’ai eu une bonne collaboration avec les pharmaciens de garde
36. Les problèmes de communication qui entrainent des retards pour s’occuper des patients sont fréquents

Answers were given on a Likert scale of 1 to 5: 1=disagree strongly, 2=disagree slightly, 3=neutral, 4=agree slightly, 5=agree strongly, N/A. Negatively worded items were reversely scored. In accordance with Sexton et al., mean scores for each of the six factors were reported on a 100-point scale using this formula: (mean score of the items of a factor – 1)*25. (Sexton JB, Helmreich RL, Neilands TB, et al. The Safety Attitudes Questionnaire: psychometric properties, benchmarking data, and emerging research. BMC Health Serv Res. 2006;6(1):1-10.)
